# Ginsenoside Rg5 Sensitizes Paclitaxel—Resistant Human Cervical-Adeno-Carcinoma Cells to Paclitaxel—And Enhances the Anticancer Effect of Paclitaxel

**DOI:** 10.3390/genes13071142

**Published:** 2022-06-24

**Authors:** Janani Ramesh, Rejani Chalikkaran Thilakan, Raja Mohan Gopalakrishnan, Singaravel Vijayapoopathi, Arianna Dorschel, Bhuvarahamurthy Venugopal

**Affiliations:** 1 Department of Medical Biochemistry, Dr. A. L. M. Postgraduate Institute of Biomedical Sciences, University of Madras, Taramani, Chennai 600113, India or jramesh@mgh.harvard.edu (J.R.); rejanict.004@gmail.com (R.C.T.); 2 Center for Regenerative Medicine, Simches Research Center, Massachusetts General Hospital, Boston, MA 02114, USA; 3 Center for Advanced Studies in Botany, University of Madras, Guindy Campus, Chennai 600025, India; rgrajamohan@yahoo.com; 4 Department of Pharmacology and Environment Toxicology, Dr. A. L. M. Postgraduate Institute of Biomedical Sciences, University of Madras, Taramani, Chennai 600113, India; singaravel.jul@gmail.com; 5 Department of Psychology and Neuroscience, The University of St Andrews, St Andrews KY16 9AJ, UK; aridors@gmail.com

**Keywords:** paclitaxel, cervical cancer, chemoresistance, ginsenoside, GRg5, apoptosis

## Abstract

In cervical cancer chemotherapy, paclitaxel (PTX) chemoresistance has become a major difficulty, and it also affects the survival rate of numerous tumor patients. Thus, for the reversal of chemoresistance, it is imperative to develop combinatory drugs with petite or almost no side effects to sensitize cells to paclitaxel. Ginsenoside Rg5 (GRg5) may act as a chemosensitizer by reversing multidrug resistance. The present study aimed to determine the potential of GRg5 as a chemosensitizer in PTX-resistant human cervical adeno-carcinoma cell lines (HeLa cells). MTT assay was carried out to assess whether GRg5 can potentiate the cytotoxic effect of PTX in PTX- resistant HeLa cells; using flow cytometry-based annexin V-FITC assay, cellular apoptosis was analyzed; the rate of expression of the cell cycle, apoptosis and major cell-survival-signaling-related genes and its proteins were examined using RT-PCR and Western blotting technique. We found increased mRNA expression of Bak, Bax, Bid, and PUMA genes, whereas the mRNA expression of Bcl2, Bcl-XL, c-IAP-1, and MCL-1 were low; GRg5 combination triggered the efficacy of paclitaxel, which led to increased expression of Bax with an enhanced caspase-9/-3 activation, and apoptosis. Moreover, the study supports GRg5 as an inhibitor of two key signaling proteins, Akt and NF-κB, by which GRg5 augments the susceptibility of cervical cancer cells to PTX chemotherapy. GRg5 drastically potentiated the antiproliferative and pro-apoptotic activity of paclitaxel in PTX-resistant human cervical cancer cells in a synergistic mode. Moreover, in the clinical context, combining paclitaxel with GRg5 may prove to be a new approach for enhancing the efficacy of the paclitaxel.

## 1. Introduction

Ginsenoside, a phytocompound extracted from the root of *Panax ginseng*, is a well-known deterrent to cancer [1]. Various types of ginsenosides occur in nature (Rg1, Rb1, Rd, Rg3, Rg5, and Rh2), whose composition varies based on the processing and extraction method. Although most known ginsenosides have been tested to possess anticancer activity both in vitro and in vivo [2], Ginsenoside Rg5 (GRg5) has more bioavailability and sustainability than other ginsenosides [3,4,5]. Moreover, GRg5 is also an antioxidant and vasorelaxant and contains anti-inflammatory and antimicrobial properties [6]. GRg5 is synthesized through deglycosylation of ginsenoside Rb1 and dehydration of the 20th carbon of ginsenoside Rg3 (GRg3) [7]. GRg5 inhibits cell proliferation approximately four times as effectively as GRg3 [8]. An in vitro and in vivo study demonstrated that GRg5 disables multidrug resistance (MDR) through ABCB1 transporters [9]. In breast cancer chemotherapy, GRg5 exhibited fewer side effects and it did not reduce immune cells [10]. GRg5 (standalone) also showed anti-neoplastic activity through activation of apoptosis in cervical cancer treatment [11]. Thus, the synergistic action of ginsenosides with conventional cancer therapies has been suggested to maximize its efficacy and reduce its probable side effects as well [12].

Cervical cancer is predominantly lethal to women. Mostly, certain strains of human papillomavirus (HPV) contribute to the initial cause of infection that leads to cervical cancer [13]. In developing countries, including India, 55% of deaths have been reported among women diagnosed with cervical cancer/year [14]. The high mortality is due to failure in diagnosis at the early stage and the lack of effective therapies for the metastatic stages of cervical cancer. Surgical resection followed by the parenteral administration of chemotherapeutic agents such as paclitaxel (PTX), cisplatin, or carboplatin is an established standard treatment for cervical cancer during the advanced stage [15]. Moreover, different drug designs/formulations and objects of combination treatments have been devised, yet significant side effects and drug chemoresistance among 60–80% of patients have been reported [16].

PTX, a taxanes-based chemotherapeutic agent, is a microtubule stabilizer that interferes with the normal breakdown of microtubules during cell division [17]. As a mitotic inhibitor, PTX is used as a chemotherapeutic agent in treating patients with lung, ovarian, cervical, breast, and Kaposi’s carcinoma [18,19]. However, in cervical cancer, myelotoxicity and chemoresistance due to recurrent PTX administration impede its success [20]. To overcome the chemical resistance and safeguard the patients from high doses of a single agent that also leads to non-cancerous cytotoxicity, a combination of treatments can be considered.

In the cancer cells, NF-κB and Akt are critical for cell survival and in developing resistance against chemotherapeutics [21,22]. In clinical therapy, canonical NF-κB is selectively inhibited by using anti-inflammatory drugs such as aspirin, sodium salicylate, and dexamethasone, which suppress NFκB activation [23]. Moreover, blockage of NF-κB alone does not favor cancer regression, and thus, combination therapy has been suggested. Combinations of NF-κB inhibitors and conventional therapies such as chemotherapy and radiotherapy have shown a more effective approach [24]. Moreover, combinatorial clinical therapy with NF-κB inhibitors and inhibitors of other signaling involved in inflammation, such as AP1 and STAT3, have been highly effective for certain types of cancers [25]. Diosgenin abrogated NF-κB/STAT3 activation by suppressing protein kinases and reporter gene activity that reduced various tumorigenic gene products expression [26]. Thidiazuron blocked invasion, and EMT in non-tumorigenic BEAS-2B epithelial cells stimulated with TGF-β/TNF-α and prevented EMT and blocked metastasis of breast cancer cells [27].

Ginsenoside Rg5 is known to be an ideal chemosensitizer for various types of chemotherapeutic drugs in cancer treatment by augmenting the efficacy of chemo drugs. However, the mechanism underlying the chemosensitivity of GRg5 against PTX-resistant human cervical cancer cells and as a combinatory drug for potential targeted therapy has not been investigated yet. In particular, the key targets of GRg5 and its molecular mechanisms that boost the antiproliferative activity of PTX in chemo-resistant cervical cancer cells need to be explored. In the current study, we have investigated the effects of GRg5 and PTX combination treatment on a PTX-resistant human cervical cancer cell line (HeLa-PTX-R). The main objective of this study is to trigger synergistic activity that arises from two agents, PTX and GRg5, with different molecular mechanisms having non-overlapping toxicity profiles.

## 2. Materials and Methods

### 2.1. Chemicals and Reagents

Ginsenoside Rg5, paclitaxel, and MTT [3-(4, 5-dimethylthiazole-2-yl)-2,5-diphenyl-tetrazolium bromide] were purchased from Sigma Aldrich (St. Louis, MO, USA). Low glucose Dulbecco’s Modified Eagle’s Medium (DMEM), Fetal Bovine Serum (FBS), penicillin, and streptomycin were purchased from Gibco Life Technologies (Grand Island, NY, USA). Annexin V-FITC Apoptosis Detection Kit was purchased from BD Biosciences (Franklin Lakes, NJ, USA). NF-κB, Bax, Bak, Caspase-9, Akt, Phospho-Akt, IKK-α, GAPDH, and Bcl-2 antibodies used in the study were obtained from Cell Signaling Technology (Danvers, MA, USA) and Santa Cruz Biotechnology (Dallas, TX, USA).

### 2.2. Cell Culture and Treatment

A Chemo-naive (HeLa cells) and paclitaxel-resistant human cervical cancer cell lines (HeLa-PTX-R cells) were both obtained from American Type Culture Collection (ATCC) (Boston, MA, USA). Cells were cultured in DMEM supplemented with 10% FBS, 50 μg/mL penicillin, and streptomycin at 37 °C in a 5% CO_2_ humidified atmosphere. For the initial screening assays, to determine the best synergetic combination, the cells were treated with varied concentrations of GRg5 (0, 5, 10, 20, 25, 30 μM) and PTX (0, 0.5, 1, 2, 4, 8 nM) for 24, 48, and 72 h. Then for all the further experiments, the cultured cells were treated either with PTX alone (4 nM as fixed concentration), GRg5 alone (20 μM as fixed concentration), or GRg5 (20 μM) with PTX (4 nM) as a combination. The control group, HeLa-PTX-R cells, i.e., the untreated condition, were used to normalize the treatment effects in cell proliferation assessments and other experiments discussed below. For the MTT assay, 1 × 10^4^ cells/well were plated in 96-well plates. For the Western blot, immunostaining, and other assays, cells were seeded at the rate of 2 × 10^4^ cells per 60 mm plate.

### 2.3. MTT Assay

The proliferative or cytotoxic effect of PTX and GRg5 was determined by MTT assay. The cells (1 × 10^4^/well) were seeded into 96-well plates and treated with GRg5 (0, 5, 10, 20, 25, 30 μM) and PTX (0, 0.5, 1, 2, 4, 8 nM) for 24, 48, and 72 h. In order to measure cell viability, the cells were further incubated with MTT solution (0.5 mg/mL) for an additional 4 h at 37 °C and measured the absorbance using a microplate reader (Bio-Rad Laboratories Inc., Irvine, CA, USA) at a wavelength of 490 nm.

### 2.4. Colony Formation Assay (CFA)

In order to compare the cell proliferation of each treatment group, we performed colony formation assay (CFA). Cells were counted using Hemocytometer (Sigma Aldrich, St. Louis, MO, USA), and 300 cells were seeded per well of 12-well plates (in triplicate). Fresh DMEM medium was used and replaced every 3 days. Colonies were counted on either 12 days (HeLa-PTX-R cells) or 15 days after seeding, only if they contained more than 50 cells. The cells were stained using crystal violet, and the colony formation was quantified using the colony formation number.

### 2.5. DAPI Staining Assay

In order to determine the nuclear morphological changes and apoptotic cell death, cells were stained with 4, 6-diamidino-2-phenylindole dihydrochloride (DAPI). The cells were seeded in 6-well plates and treated with vehicle control DMSO (0.01%), PTX alone (4 nM), GRg5 alone (20 μM), or GRg5 (20 μM) with PTX (4 nM) as a combination for 48 h. For the nuclear analysis, the monolayer of cells was washed with PBS and stained with DAPI (0.5 μg/mL) for 5 min. The apoptotic nuclei (intensely stained, fragmented nuclei, and condensed chromatin) were observed under a fluorescent microscope (Nikon Eclipse-80i, Nikon Instruments Inc. Melville, NY, USA).

### 2.6. Annexin V-FITC Staining for Apoptosis Assay Using Flow Cytometry

HeLa-PTX-R cells were treated with either PTX (4 nM) or GRg5 (20 μM) alone or with both in combination for 48 h. Cells were harvested, centrifuged, and the obtained pellets were washed and resuspended in binding buffer. Then, phosphatidylserine externalization was visualized by staining the suspended cells with Annexin V (antibody) conjugated with dye (Santa Cruz, Santa Cruz Biotechnology Inc. Dallas, TX, USA) according to the manufacturer’s instructions, and photomicrographs were taken. Unstained cells were used as negative control. The stained cells (Annexin V-FITC) were plotted against SSC-H to analyze the apoptotic cells. Flow cytometry data were analyzed using BD FACS Diva software, version 5.0.2 (BD Biosciences, San Jose, CA, USA).

### 2.7. Determination of Gene Expression by the RT-PCR

HeLa-PTX-R cells were treated with either PTX (4 nM) or GRg5 (20 μM) alone, or in combination (GRg5 20 μM + PTX 4 nM) for 48 h. Following treatment, RNA was isolated using an RNA isolation kit (Roche Diagnostics GmbH, Mannheim, Germany). About 2 μg of RNA from each group was used to construct the corresponding cDNA (SuPrime Script RT Premix, 2X) as per the manufacturer’s instruction (Roche Diagnostics GmbH, Mannheim, Germany). Specific sets of primers (Eton Biosciences, San Diego, CA, USA) with SYBR green master mix were used for the reaction to verify gene expression (Table 1).

For the qRT-PCR reaction, the final volume used was 10 µL which includes 0.05 µL of each 10 µM forward and 10 µM reverse primer, 5 µL of 2× SYBR Green PCR master mix, and 2 µL of 1:10 diluted cDNA solution. Melting curve analysis was performed for each primer with annealing temperature steadily from 60 °C to 90 °C, and the temperature was raised by 0.5 °C by each 5 s.

The relative mRNA expression of Bak, Bax, Bid, PUMA, Bcl-XL, Bcl-2, c-IAP-1, and MCL-1 were determined by RT-PCR. The rates of variations in gene expression were evaluated in terms of fold change to the untreated cellular population (control) by 2-∆∆CT method. An initial denaturation phase at 95 °C for 10 min was followed by further denaturation (95 °C, 15 s), annealing (different annealing temperatures for each primer, 10 s), and final extension (2 °C, 5 min).

### 2.8. Western Blot Analysis

HeLa-PTX-R cells were treated with either PTX (4 nM) or GRg5 (20 μM) alone or with both in combination (GRg5 20 μM + PTX 4 nM) for 48 h. Cells were washed, homogenized using lysis buffer (50 mM Tris pH 8.0, 150 mM NaCl, 0.02% sodium azide, 0.2% SDS, 1 mM PMFS, 10 μL/mL aprotinin, 1% igapel630, 10 mM NaF, 0.5 mM EDTA, 0.1 mM EGTA and 0.5% sodium deoxycholate), and centrifuged at 23,000× *g* for 1 h. Bradford’s method (Bio-Rad Protein Assay, Bio-Rad Laboratories, Hercules, CA, USA) was used to measure protein concentration. Equal amounts of protein (50 μg/well) were separated in a 12% SDS polyacrylamide gel, transferred to a nitrocellulose membrane (Hybond ECL, Amersham Pharmacia Biotech, Piscataway, NJ, USA), and blocked with 5% (*w*/*v*) non-fat dried milk in Tris-buffered saline solution (10 mM Tris, pH 8.0, 150 mM NaCl) containing 0.05% tween-20. The membranes were incubated with rabbit polyclonal primary antibodies Bax, Bak, t-Akt, and p-Akt (1:2000 µL dilution, Santa Cruz Biotechnology, Santa Cruz, CA, USA), caspase-9, cleaved caspase-9, Bcl-2, NF-κB, and IKK-α (1:3000 µL dilution, Cell Signaling Technology, Beverly, MA, USA) which were incubated with the corresponding anti-rabbit or anti-mouse secondary antibodies (1:5000 µL dilution, Santa Cruz Biotechnology, CA, USA). An ECL was performed for the relative densities of the protein bands using the MyImage software system and quantified by Lab works 4.0 software (UVP Inc., Upland, CA, USA). At least 3 independent experiments in each case were performed and normalized with GAPDH protein.

### 2.9. Statistical Analysis

All statistical analyses were performed using Graph pad Prism (version 8.03; Graph pad Software, San Diego, CA, USA). For comparisons between control and treatment, one-way analysis of variance (ANOVA) followed by Tukey’s post hoc test for multiple comparisons were used. Data were analyzed and presented as mean± S.E.M and considered *p*-values ≤ 0.0001 of extremely high significance (indicated by ****); *p*-values ≤ 0.001, high significance (indicated by ***); *p*-values ≤ 0.01, moderately significant (indicated by **); and *p*-values ≤ 0.05, low significance (indicated by *); and *p*-values > 0.05, non-significant (n.s).

## 3. Results

### 3.1. Efficacy of GRg5 Combined Paclitaxel Treatment on Paclitaxel Chemo Resistant Cervical Cancer Cells (HeLa PTX—R Cells)

#### 3.1.1. Cytotoxic Effect of GRg5, PTX, and Combination of GRg5 and PTX in HeLa-PTX-R Cells

**Figure 1:**
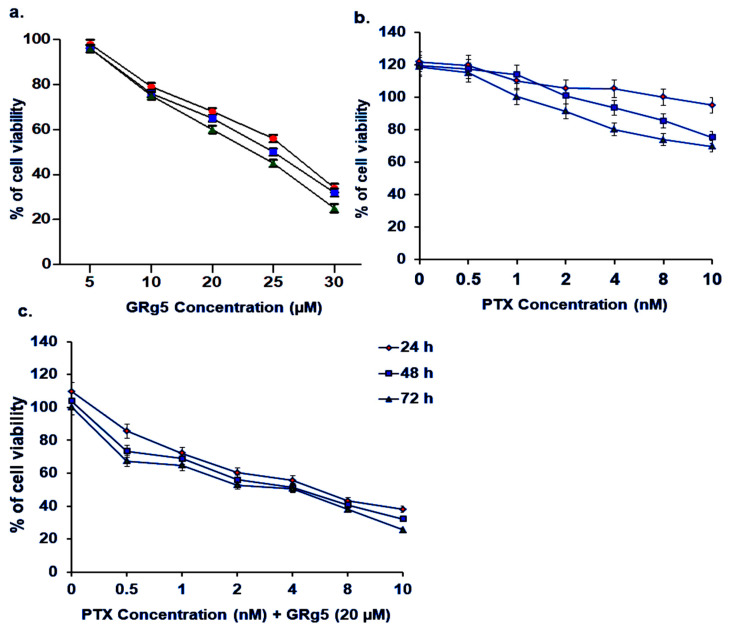
**MTT cell proliferation assay:** The representative line diagrams showing the cell viability of HeLa-PTX-R cells after treatment with (**a**) GRg5 (5, 10, 20, 25, 30 µM) with varied concentration for initial screening (**b**) PTX (0, 0.5, 1, 2, 4, and 8 nM) and (**c**) GRg5 20 μM combined with PTX (0, 0.5, 1, 2, 4, 8 nM) to analyze the best synergetic combination for 24 h, 48 h and 72 h. The absorbance of the samples was measured at a wavelength of 490 nm. Each value is mean ± S.E.M of three individual experiments and the data was normalized with the control group.

To evaluate the cytotoxic effect (IC50) of GRg5, PTX, and GRg5+PTX combination, HeLa-PTX-R cells were treated with different concentrations of GRg5 (0, 5, 10, 20, 25, 30 μM) and PTX (0, 0.5, 1, 2, 4, 8 nM) and GRg5 (20 μM)+PTX (0, 0.5, 1, 2, 4, 8 nM) combination for 24, 48, and 72 h. The results of the MTT assay revealed that HeLa-PTX-R cells with 10, 20, and 30 μM treatment of GRg5 moderately reduced the rate of cell viability (30–60%) (Figure 1a), while 4, 8, 10 nM treatment of PTX to these cells also reduced the rate of cell viability, albeit 20–25% only (Figure 1b). Interestingly, in the combination treatment, the cell viability was reduced to 50–75% (Figure 1c). Furthermore, the IC50 value (20 μM) of GRg5 that we choose is also equivalent to the previously reported study by Liang et al., 2015 [9].

**Figure 2:**
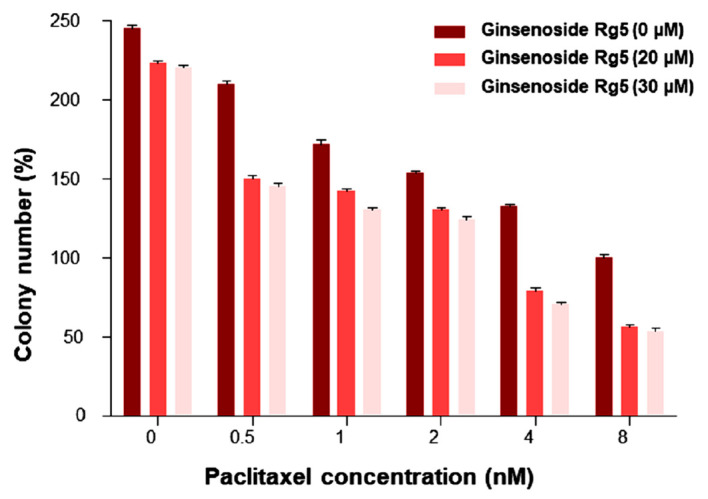
**Colony Formation Assay:** Representative bar diagram is the colony number data for HeLa-PTX-R cells treated with various concentrations of GRg5 (0, 20, and 30 μM) combined with PTX (0, 0.5, 1, 2, 4, and 8 nM) to analyze the best synergetic combination. Each value is mean ± S.E.M of three individual experiments.

In order to validate the cell proliferation, a colony formation assay (CFA) was performed. (HeLa-PTX-R cells were treated with various concentrations of GRg5 (0, 20, 30 μM) and PTX (0, 0.5, 1, 2, 4, 8 nM). The performed CFA exhibited diminution in colony formation in a dose-dependent manner (Figure 2). Colony formation was much pronounced with a reduction in the cells with treatment of GRg5 (20 μM) +PTX (4 nM) and GRg5 (30 μM) +PTX (4 nM), compared to the other concentrations having combinations of GRg5 and PTX.

**Figure 3:**
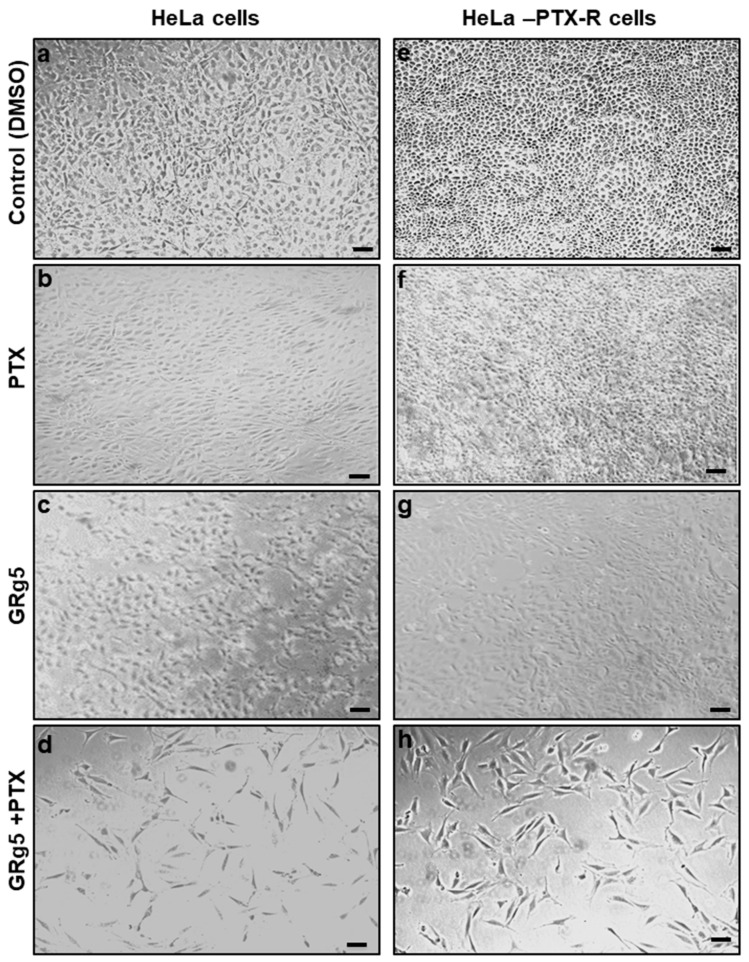
**Cytotoxic effects of GRg5 on HeLa and HeLa-PTX-R cells:** Representative photomicrographs showing morphological changes and cell viability of HeLa naive (**a**–**d**) and HeLa-PTX-R cells (**e**–**h**) cervical cancer cells after treatment with GRg5 (20 μM) only, PTX (4 nM) only or co-treatment with 20 μM of GRg5 and 4 nM of PTX for 48 h.

In order to characterize the resistance (IC50) of HeLa-PTX-R cells and chemo-naive HeLa cells, the rate of cell viability was also assessed using cell morphology assessments. The cell viability assessment of hemo-naive HeLa cells and HeLa-PTX-R cells confirmed the PTX chemo resistance property of HeLa-PTX-R cells (Figure 3a,e). In addition, cell viability assays of HeLa-PTX-R cells also revealed that an adequate dose of GRg5 (20 μM) required to chemosensitize cells and overcome PTX drug resistance. It also showed diminished viability (*p* < 0.001) of the HeLa-PTX-R cells with the combination treatment [PTX (4 nM) + GRg5 (20 μM)] compared to GRg5 (20 μM) alone (Figure 3a–d), we have also tested the PTX drug alone and combination with GRg5 in normal Hela cells, that too confirmed the good efficacy of PTX in treating cancer cells when combined with GRg5 (Figure 3e–h). The IC50 value of HeLa-R cells for paclitaxel was much higher than in HeLa cells, indicating approximately 15–20-fold greater resistance.

#### 3.1.2. Effect of GRg5 in PTX Induced Apoptosis in HeLa-PTX-R Cervical Cancer Cells

**Figure 4:**
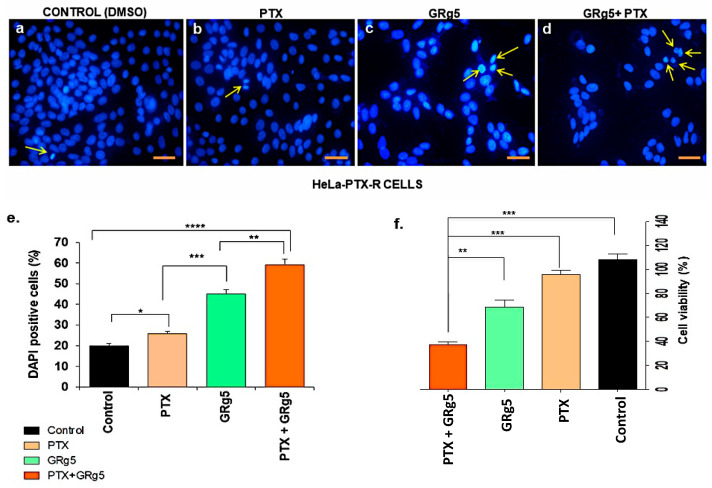
**Detection of apoptosis by DAPI staining:** Representative fluorescent photo micro-graphs showing apoptotic cells through DAPI staining of (**a**) control HeLa-PTX-R cells (DMSO), (**b**) cells treated with PTX alone, (**c**) cells treated with GRg5 alone, and (**d**) cells treated with PTX+GRg5. Histogram of quantitative analysis of (**e**) apoptotic cells (brightly fluorescent indicated with yellow arrow), and (**f**) the rate of cell viability. Three independent experiments were conducted. Data is represented as mean ± S.E.M of three individual observations. Statistical significance was assessed using One way-ANOVA and Tukey’s post hoc test. ****, *p* < 0.0001; ***, *p* < 0.001; **, *p* < 0.01; *, *p* ≤ 0.05. Scale bar = 100 μm.

In order to validate the morphological changes of chromatin and cell death, DAPI staining was performed, which exhibited maximum reduction (70–80% reduction in the rate of cell viability) of cervical cancer cells when GRg5 and PTX were used in combination. There was a diminution of 40–50% of cervical cancer cells with treatment of GRg5 alone, while it was only a 10–15% reduction with the treatment of PTX alone (Figure 4a–e).

**Figure 5:**
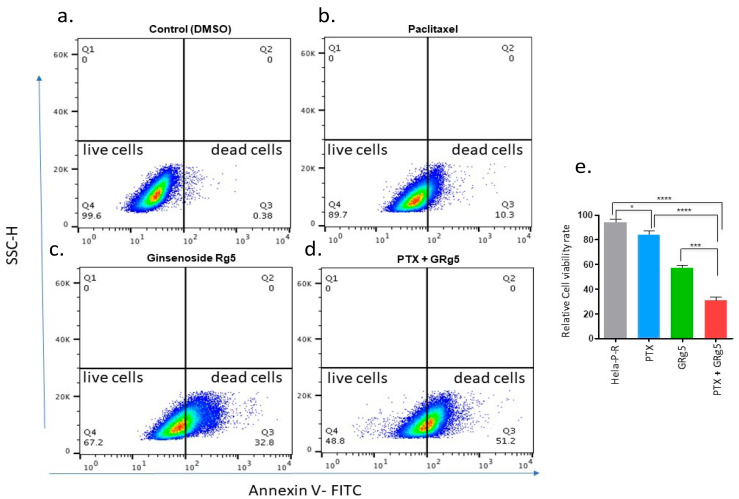
**Flow cytometry analysis of HeLa-PTX-R cells by using Annexin V-FITC/PI staining:** The cytotoxicity of HeLa-PTX-R cells was evaluated, and the representative photographs shows the live and dead cell population of Hela PTX-R cells of (**a**) untreated (**b**) PTX alone (4 nM) treated cells (**c**) GRg5 alone (20 µM) treated cells and (**d**) GRg5 (20 µM) + PTX (4 nM) combined treated cells. The X and Y axis (a-d) represents the intensity of FITC Annexin V and SSC-H, respectively. The gated Quadrant 4 (Q4) is Annexin V-FITC negative cells indicating live cell population, and Quadrant 3 (Q3) is Annexin V-FITC positive cells indicating dead cell population. The graph plot (**e**) represents the percentage of viable cells. Data is represented as mean ± S.E.M of three individual observations. Statistical significance was assessed using One way-ANOVA and Tukey’s post hoc test. ****, *p* < 0.0001; ***, *p* < 0.001; *, *p* ≤ 0.05.

In order to evaluate apoptosis quantitatively, an Annexin-V-FITC staining assay was performed in HeLa-PTX-R cells. The percentage of cells that were positive for Annexin V-FITC staining was 72% in GRg5+PTX treated cells (Figure 5a–e), indicating excessive apoptotic cell death due to combination treatment in these cells (Figure 5d). However, the rate of cell death with the treatment of GRg5 alone (Figure 5c) or PTX alone was only 40% and 10–20%, respectively (Figure 5b).

#### 3.1.3. Effect of GRg5 on mRNA Expression of Apoptotic Regulatory Genes in PTX Treated HeLa-PTX-R Cell Lines

**Figure 6:**
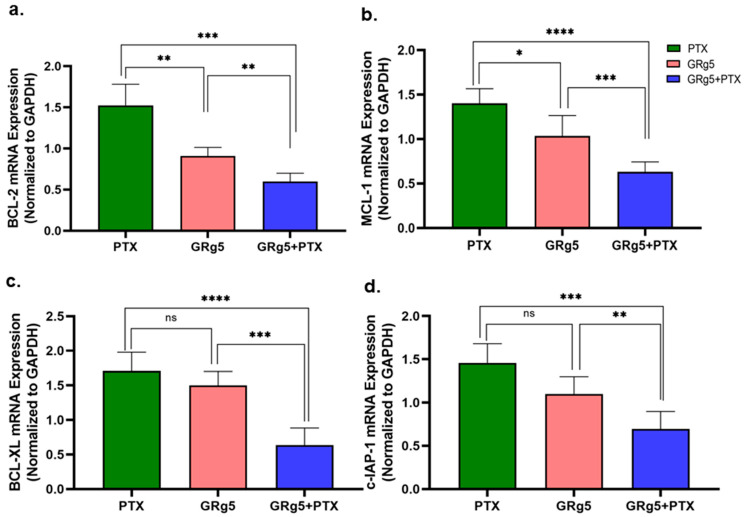
**Quantitative RT-PCR analysis of anti-apoptotic regulatory genes:** The mRNA expression of anti-apoptotic genes (**a**) Bcl-2, (**b**) MCL-1, (**c**) Bcl-xL, and (**d**) cIAP-1 in control and treated HeLa-PTX-R cells quantified relative to the control and normalized against the reference gene, GAPDH. Data is represented as mean ± S.E.M of three individual observations. Statistical significance was assessed using One way-ANOVA and Tukey’s post hoc test. ****, *p* < 0.0001; ***, *p* < 0.001; **, *p* < 0.01; *, *p* ≤ 0.05; ns, non-significant.

In order to validate the mRNA expression of apoptotic regulatory genes in the PTX-treated cells, key pro-apoptotic and anti-apoptotic genes were investigated and analyzed by RT-PCR. The mRNA expression of pro-apoptotic genes Bak (Figure 6a), Bax (Figure 6b), Bid (Figure 6c), and PUMA (Figure 6d) were augmented significantly in the cells with combined treatment of GRg5 and PTX when compared to control and GRg5 alone treated cells (Figure 6).

**Figure 7:**
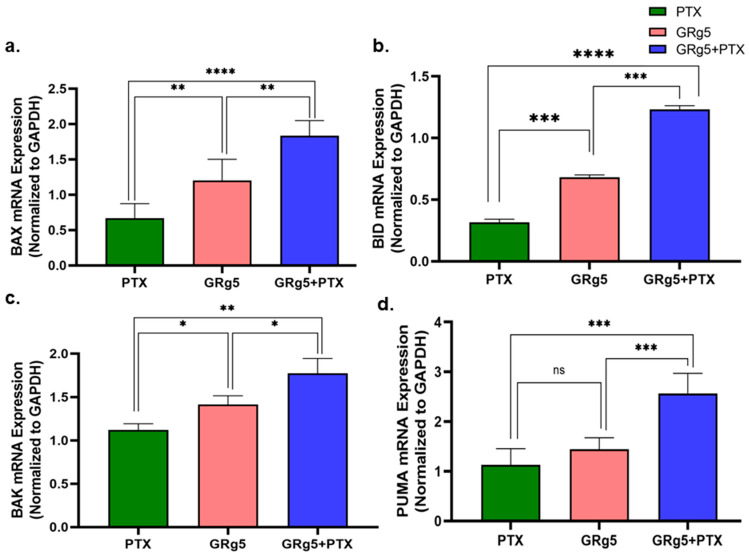
**Quantitative RT-PCR analysis of pro-apoptotic regulatory genes:** Expression of different pro-apoptotic genes (**a**) Bax, (**b**) BID, (**c**) BAK, and (**d**) PUMA in control and treated HeLa-PTX-R cells quantified relative to the control and normalized against the reference gene, GAPDH. Data is represented as mean ± S.E.M of three individual observations. Statistical significance was assessed using One way-ANOVA and Tukey’s post hoc test. ****, *p* < 0.0001; ***, *p* < 0.001; **, *p* < 0.01; *, *p* ≤ 0.05; ns, non-significant.

On the contrary, the mRNA expression of anti-apoptotic genes Bcl-XL (Figure 7a), Bcl-2 (Figure 7b), c-IAP-1 (Figure 7c), and MCL-1 (Figure 7d) were diminished in the cells with either GRg5 alone treatment or the combination of GRg5 and PTX, compared to control (Figure 7). However, the effect was much more pronounced in the cells with combination treatment.

#### 3.1.4. Effect of GRg5 on Apoptotic Regulatory Proteins in PTX Treated HeLa-PTX-R Cell Lines

**Figure 8:**
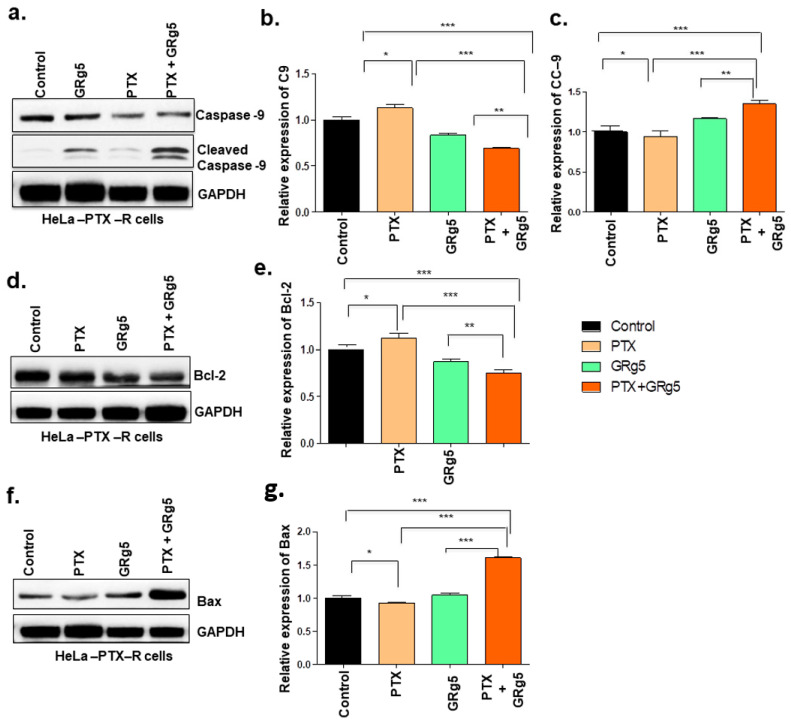
**Quantitative analysis of apoptotic regulatory proteins expression levels using Western Blot:** HeLa-PTX-R cells, with treatment of GRg5 alone, PTX alone, or in combination with GRg5 (20 μM) + PTX (4 nM) for 48 h, were investigated and detected by Western blot for the apoptosis regulatory proteins. The figure (**a**) caspase 9 and cleaved caspase 9, (**d**) Bcl2, (**f**) Bax represents the blots. The graph plot (**b**,**c**,**e**,**g**) represents the quantitative analysis of respective blots of apoptotic regulatory proteins. Data is represented as mean ± S.E.M of three individual observations. Statistical significance was assessed using One way-ANOVA and Tukey’s post hoc test. ***, *p* < 0.001; **, *p* < 0.01; *, *p* ≤ 0.05.

In order to determine whether GRg5 can promote PTX-induced activation and/or suppression of apoptotic regulatory proteins, the key proteins associated with it were detected by Western blot using whole-cell extracts of HeLa-PTX-R cells treated with GRg5 (20 μM) and PTX (4 nM). Western blot data along with histogram data of caspase 9 and cleaved caspase 9, (Figure 8a–c) Bcl2 (Figure 8d,e), and Bax (Figure 8f,g) showed the less expression of anti-apoptotic Bcl-2 protein, whereas pro-apoptotic proteins Bax and cleaved caspase-9 (the active form of caspase-9) showed augmented expression in the cells with GRg5+PTX combination treatment, compared to control.

#### 3.1.5. Effect of GRg5 on Cell Cycle Regulatory Proteins in PTX Treated HeLa-PTX-R Cell Lines

**Figure 9:**
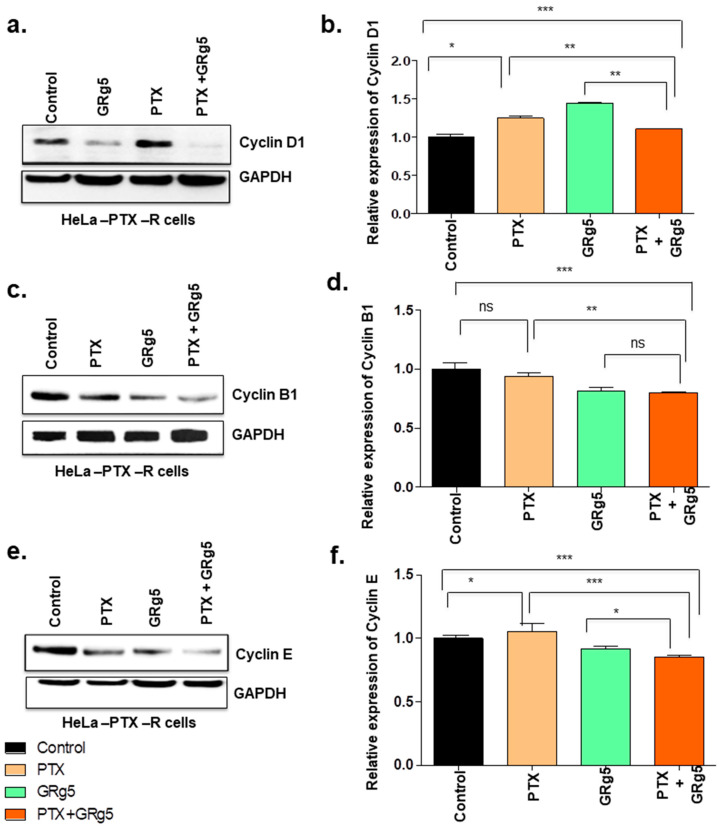
**Quantitative analysis of cell cycle regulatory proteins expression levels using Western Blot:** HeLa-PTX-R cells, treated with GRg5 alone, PTX alone or in combination with GRg5 (20 μM) + PTX (4 nM) for 48 h, were investigated. The figures represent the Western blot of proteins (**a**) cyclin D1, (**c**) cyclin B1, and (**e**) cyclin E. The corresponding histogram (**b**,**d**,**f**) represents the quantitative analysis of the blot of each cell cycle regulatory proteins. Data is represented as mean ± S.E.M of three individual observations. Statistical significance was assessed using One way-ANOVA and Tukey’s post hoc test. ***, *p* < 0.001; **, *p* < 0.01; *, *p* ≤ 0.05; ns, non-significant.

We were interested to know the effect of GRg5 and its combination treatment with PTX on the cell-cycle-specific proteins as the progression of the cell cycle is known to be regulated by several cyclins and CDK activities. We investigated key cell cycle mediating proteins cyclin D1, B1, E, CDK-2, and CDK-4. Western blot detection revealed decreased levels of cyclin D1, B1, and E in the cells with GRg5+PTX combination treatment, compared to control (Figure 9a–f).

**Figure 10:**
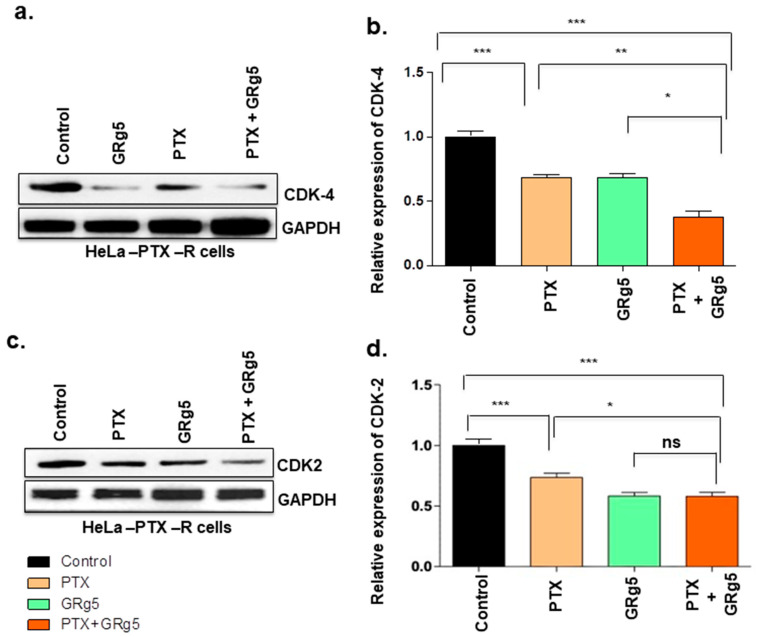
**Quantitative analysis of cell cycle regulatory CDK proteins expression levels using Western Blot:** HeLa-PTX-R cells, treated with GRg5 alone, PTX alone or in combination with GRg5 (20 μM) + PTX (4 nM) for 48 h, were investigated. The figures represent Western blot of (**a**) CDK4, and (**c**) CDK2 proteins. The graph plot (**b**,**d**) represents the quantitative analysis of the blots. Data is represented as mean ± S.E.M of three individual observations. Statistical significance was assessed using One way-ANOVA and Tukey’s post hoc test. ***, *p* < 0.001; **, *p* < 0.01; *, *p* ≤ 0.05; ns, non-significant.

Interestingly, cyclin B1 protein showed comparable outcomes in GRg5 alone and GRg5+PTX combination treatment. Moreover, cyclin-dependent kinases, CDK4, and CDK2 proteins also decreased in the cells with combined treatment of GRg5+PTX compared to control (Figure 10a–d). However, GRg5 alone treatment was comparable with the combined treatment of GRg5+ PTX for CDK-2 protein, but it was less effective on CDK4 protein.

#### 3.1.6. Effect of GRg5-PTX Combination Treatment on Akt/IKK-α/NF-κB Signaling Pathways

**Figure 11:**
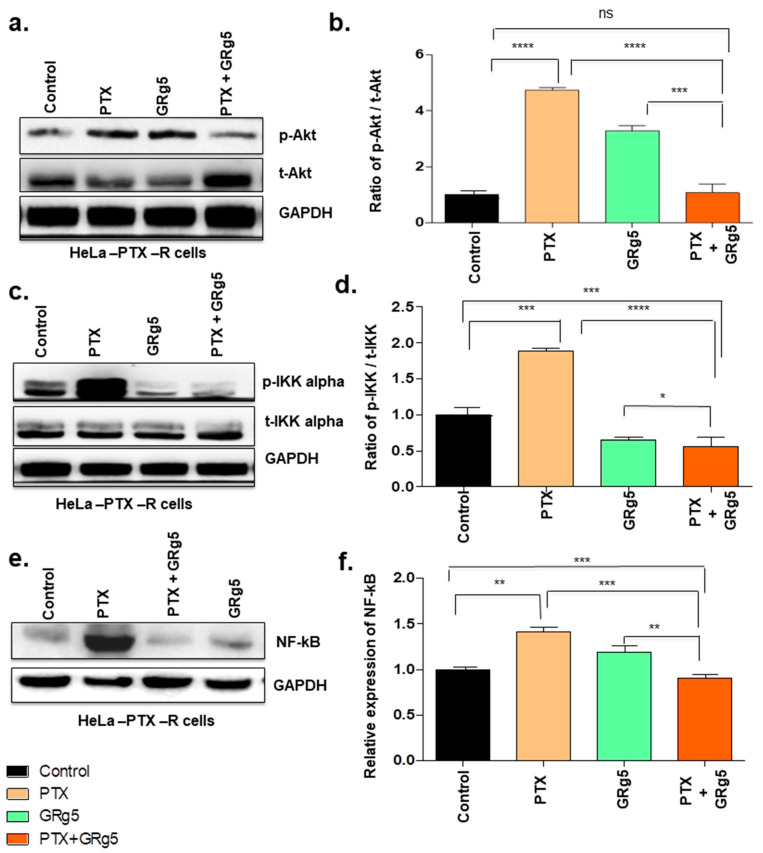
**Quantitative analysis of cell survival signaling proteins expression levels using Western Blot analysis:** HeLa-PTX-R cells, treated with GRg5 alone, PTX alone or in combination with GRg5 (20 μM) + PTX (4 nM) for 48 h, were investigated. The figures represent Western blot of proteins (**a**) p-Akt, (**c**) p-IKK-α, (**e**) NF-κB. The graph plot (**b**,**d**,**f**) represents the blot quantitative analysis. Data is represented as mean ± S.E.M of three individual observations. Statistical significance was assessed using One way-ANOVA and Tukey’s post hoc test. ****, *p* < 0.0001; ***, *p* < 0.001; **, *p* < 0.01; *, *p* ≤ 0.05; ns, non-significant.

Since the activation of NF-κB and Akt are critical for cancer cell survival and in developing resistance against chemotherapeutics, the effect of GRg5 on Akt and NF-κB signaling molecules was investigated. The result showed increased p-Akt (activated form of Akt) in the cells with PTX standalone treatment, whereas combined GRg5 and PTX treatment decreased p-Akt protein expression, compared to PTX or GRg5 treatment alone (Figure 11a,b). Moreover, a reduction in p-IKKα was also observed with the combined treatment of GRg5 and PTX treatment (Figure 11c,d). Western blot detected diminished NF-κB protein level (Figure 11e,f) when the HeLa-PTX-R cells were treated in combination with GRg5+PTX as compared to control, PTX alone, or GRg5 alone treatment (*p* < 0.001).

## 4. Discussion

Chemoresistance to chemotherapies is a colossal issue in treating various cancers. Many chemotherapeutic agents become ineffective after their consistent use [28]. Taxol, an effective chemotherapeutic drug, is used to treat different types of cancers, e.g., cervical, breast, and ovarian cancer, but it also develops resistance due to consistent use [29]. Paclitaxel (PTX), a first-line Taxol-based chemo drug, is extensively used to treat various types of cancers, including lung cancer, breast cancer, cervical cancer, etc. [30]. It inhibits microtubule growth and promotes the polymerization of tubulin, which is used for the treatment of cervical cancer [31]. However, during chemotherapy, patients often develop chemoresistance to PTX, and thus mono-drugs with high doses bear the risk of drug-induced toxicity [32]. Consistent use of PTX drug becomes paclitaxel-resistant and does not reduce the rate of cell proliferation, and contributes to the consecutive recurrence and metastasis of cancer, ultimately causing death [33]. In order to lessen the effect of drug resistance, many cancer therapeutics with combination treatments such as the addition of phytochemicals to Taxol (PTX) are considered [34]. It has been demonstrated that GRg5 exhibits improved pro-apoptotic effects on human breast cancer cell lines compared to ginsenoside Rg3. A recent study showed that GRg5 induced breast cancer cell apoptosis and autophagy by inhibiting the PI3K/Akt/mTOR signaling pathway [10]. Additionally, GRg5 promotes apoptosis in retinoblastoma cells by inhibiting the Akt signaling pathway and thereby downregulates Bcl-2 expression. GRg5 also promotes human cervical cancer cell apoptosis by inducing DNA double-strand breaks and fragments. The above studies clearly suggest the efficacy of GRg5 as a good anticancer agent, and hence we choose this compound as a chemosensitizer for the synergetic mechanism with PTX to evade cancer cells that are resistant to PTX. In the current study, we have demonstrated that GRg5 (a compound of ginseng root extract) enhances PTX-mediated anti-neoplastic efficacy and potentially reduces drug resistance in PTX-drug-resistant cervical cancer cells. Many phytocompounds such as curcumin, resveratrol, and quercetin have been reported to have an antiproliferative activity that enhances the effect of various chemotherapeutic agents, mainly taxanes in a range of cancer cells [35,36]. Phytochemical phenoxodiol, a synthetic derivative of genistein, sensitizes chemo-resistant ovarian cancer cells treated with platinum, taxanes, gemcitabine, and topotecan [37]. In mice with ovarian tumors, a combination of ginsenoside Rg3 with cyclophosphamide augmented the survival rate [38]. These pieces of evidence thrust on the use of combination treatments with chemotherapeutic drugs, and the current study shows the alignment with them.

Apoptosis is a unique form of cell death and is an important process that regulates the homeostasis of cell survival [39]. Apoptosis eliminates potentially cancerous cells, and this process is caused by atrophy of cells, synthesis of new proteins, and cell suicide [40]. Ginsenoside GRg5 is known to intensely reduce the proliferation of cervical cancer cells by activating apoptosis and autophagy [11]. We observed the expression status of Bcl-2 protein families, which are the important initiator of the intrinsic mitochondrial apoptotic pathway. Bcl-2 protein family includes both the pro-apoptotic proteins (Bax and Bad) and the anti-apoptotic protein (Bcl-2) [41]. Bcl-2 and Bcl-xL can stimulate certain signaling pathways, which provokes mitochondrial cells to sustain in an anti-apoptotic signaling mode during a chemotherapeutic treatment [42]. Moreover, the overexpression of Bax, a pro-apoptotic protein, promotes mitochondrial outer membrane permeabilization, then activates the caspases [43]. In the present study, a decrease in the mRNA levels of anti-apoptotic genes Bcl-2, Bcl-xL, c-IAP-1, and MCL-1 and a concomitant increase in pro-apoptotic genes (Bax, Bak, BID, and PUMA) and decreased the ratios of Bcl-2/ Bax, Bcl-xL/Bax, and MCL-1/Bax, suggest the occurrence of apoptosis in these cells. Further, the study also showed an alteration in the status of Caspase-9, a key protein involved in initiating of cell death intrinsic pathway [44]; when GRg5 combined with PTX and promoted the cleavage of caspase 9. The results indicate that GRg5, in combination with PTX, augments Bax and cleaved caspase-9 and simultaneously diminishes Bcl-2, which confirms the efficacy of GRg5 aided PTX to control PTX resistant cervical cancer cells through a mitochondrial-mediated intrinsic apoptotic pathway. In addition, the results also indicate that GRg5 combined treatment enhances both the protein and mRNA levels compared to PTX or GRg5 standalone treatment.

In the cell cycle, the G1−S transcriptional network is involved in two crucial aspects of cell cycle regulation, cell division cycle control and maintenance of genome stability. Phosphorylation of transcriptional inhibitors by cyclin-dependent kinase (CDK) activates G1–S genes [45], while cyclin D–CDK4 and cyclin D–CDK6 release them from the E2F transcription factors and induces the transcription of G1–S target gene, including cyclin E. Cyclin D1 also regulates apoptosis which can be pro- or anti-apoptotic, depending on the proliferative and differentiated state of the cell [46]. CDK4 is reported to be involved in cervical tumorigenesis, and 72.6% of cervical cancer specimens showed overexpression of CDK4 [47]. The release of cells from G1 phase arrest and its entry into the S-phase is mediated through pRb phosphorylation and inactivation by the cyclin D1/CDK4 complex. Moreover, cyclin E, with its catalytic partner CDK2 regulates the G1 phase of the cell cycle [48]. CDK2 possesses an apoptosis-sensitizing effect and arrests the cell cycle at different stages. In the present study, the cyclin D1, CDK4, cyclin E, and CDK2 status diminished in the cells with combination treatment of GRg5 and PTX, suggesting the synergistic effect of the drug may have arrested the cell division at the G1 phase. Cyclin B forms a complex with CDK1 and regulates the transition from G2 to the M phase [49]. p53 is known to regulate the G2 checkpoint through cyclin B and prevents the G2/M transition by decreasing the level of cyclin B protein [50]. The results of the present study showed diminution of cyclin B protein in the cells with combined treatment of GRg5 and PTX, suggesting the cell cycle may have arrested at the G2 checkpoint as well.

Akt signaling pathway is crucial for the regulation of cell proliferation, apoptosis, differentiation, tumorigenesis, and angiogenesis in cervical cancer [51]. It also plays a major role in the development of taxanes-based chemoresistance in cancers by regulating the factors responsible for cell proliferation and promoting the inhibition of apoptosis [52]. Akt is involved with the proliferation of various cancers [53]. Phytochemical resveratrol sensitizes the docetaxel-resistant breast cancer cells through downregulation of the PI3K/Akt pathway [54]. Moreover, a study in human esophageal cancer cells showed that GRg5 facilitated apoptosis via the phosphoinositide-3 kinase/protein kinase B signaling pathway [14]. Moreover, in the current study, the results suggest the synergetic effects between GRg5 and PTX drugs in reducing cancer cell viability could be due to inhibition of p-Akt protein expression, which is not seen in PTX or GRg5 alone treatment. Overall, the results suggest that GRg5 potentiated the effects of PTX, which induced Akt downregulation. This, in turn, diminished p-IKK-α protein in GRg5 combined PTX treatment, a condition that facilitates augmented apoptosis rates in HeLa-PTX-R cells by downregulating the major proteins involved in survival signaling pathways. The Akt pathway also regulates NF-κB activation by IKK-α dependent mechanism, which is crucial in cell survival maintenance by inhibiting apoptosis [23]. In our study, the combination treatment (GRg5 with PTX) induced significant cell death in cervical cancer cells and appeared to be mediated by the downregulation of the Akt pathway along with the downregulation of the IKK-α /NF-κB pathway. Furthermore, the adverse negative regulation of the NF-κB and Akt pathway in the combination treatment and the phosphorylation pattern of Akt by each standalone treatment and their combination was similar to that of IKK-α. Overall, the current study elucidates that Akt plays a crucial role in paclitaxel resistance treatment by upstream regulation of the IKK-α dependent mechanism, invoking the effect of ginsenoside in augmenting PTX-induced apoptosis.

We investigated the effect of GRg5 combined PTX treatment in the IKK-α/NF-κB pathway since studies have previously revealed that the Akt pathway regulates NF-κB activation by IKK- α dependent mechanism, which is crucial in cell survival maintenances by inhibiting apoptosis [55,56]. We find that combination treatment (GRg5 with PTX) induced significant cell death in cervical cancer cells may be mediated by the downregulation of the Akt pathway along with the downregulation of the IKK-α/NF-κB pathway. Furthermore, the adverse negative regulation of the NF-κB and Akt pathway is observed pointedly in the combination mode of treatment, and the phosphorylation pattern of Akt by each standalone treatment and their combination was similar to that of IKK-α; these all confer that these actions may be at least partly through the suppression of the IκB degradation and phosphorylation in PTX-resistant cervical cancer cells [57]. Overall, our study elucidates that Akt plays a crucial role in paclitaxel resistance treatment by upstream regulation of IKK-α dependent mechanism, invoking the effect of ginsenoside in augmenting PTX-induced apoptosis. In short, this study, for the first time, illustrates evidence for a synergistic mechanism of the potential utility of GRg5 in overcoming the chemoresistance of paclitaxel in cervical cancer cells.

## 5. Conclusions

GRg5, when combined with PTX, augments the chemosensitivity of PTX-resistant cervical cancer cells by inhibiting cell viability, proliferation, and induction of apoptosis via inhibiting the cell cycle. Further, the combinational treatment arrested the cell cycle and induced caspase-dependent apoptosis by downregulating PI3K/Akt/IKK-α/NF-κB signaling pathway leading to the inhibition of cervical cancer cells. Although we tested the drug combinations on the commonly used cervical cancer cell lines, we understand the caveat of not using more cell lines. Also, there is a need to expand the work with in vivo models to decisively reach the conclusions through our future investigations. To address these issues, the future direction is to validate this combination treatment in Patient-Derived Xenograft (PDX) cervical cancer in mice models. Nevertheless, the current results suggest that GRg5 could be considered as a potential chemosensitizer for various types of cancers after in vivo experiments and clinical trial validations. Taken together, pharmaceutical modulation of signaling events by an efficient combinatorial therapeutic molecule with PTX may represent new therapeutic approaches in cervical cancer treatments.

## Figures and Tables

**Table 1:** List of primers used for RT-PCR.

Gene	Forward Sequence	Reverse Sequence
***BCL-2***	5′ GATGTGATGCCTCTGCGAAG 3′	5′ CATGCTGATGTCTCTGGAATCT 3′
***BCL-xL***	5′AACATCCCAGCTTCACATAACCCC 3′	5′ GCGACCCCAGTTTACTCCATCC 3′
***c-IAP-1***	5′ GTTTTAAAACCAGCTTGGGTTATATTG 3′	5′ GTTCCTCACCCAACCAGTCTACTTAG 3′
***MCL-1***	5′ CCAAGAAAGCTGCATCGAACCAT 3′	5′ CAGCACATTCCTGATGCCACCT 3′
***BAX***	5′ GGTTGTCGCCCTTTTCTA 3′	5′ CGGAGGAAGTCCAATGTC 3′
***BID***	5′ AAATAGTTTGGGGATTTTGAAT 3′	5′ AATACACTCACCACCCTCC 3′
***BAK***	5′ GGCAGGGTATGGTATGGTTG 3′	5′ TCCCGACTGCCTGGTTACTG 3′
***PUMA***	5′ AGTACATCCTCTGGGCTCTGC3′	5′ CGGACAAGTCAGGACTTGCAGG 3′
***GAPDH***	5′ GCGAGAAGATGACCCAGAT 3′	5′ GAGGCGTACAGGGATAGC 3′

## Data Availability

We author declare that the data supporting the findings of this study are available within the paper. The data are also available from the corresponding author upon request.
